# Nature-Inspired Synthesis of Yeast Capsule Replicas Encased with Silica-Vinyl Functionality: New Fluorescent Hollow Hybrid Microstructures

**DOI:** 10.3390/molecules29225363

**Published:** 2024-11-14

**Authors:** Beata Miksa, Katarzyna Trzeciak, Slawomir Kaźmierski, Artur Rozanski, Marek Potrzebowski, Krystyna Rozga-Wijas, Lukasz Sobotta, Magdalena Ziabka, Magdalena Płódowska, Karol Szary

**Affiliations:** 1 Centre of Molecular and Macromolecular Studies, Polish Academy of Science, Sienkiewicza 112, 90-363 Lodz, Poland; katarzyna.trzeciak@cbmm.lodz.pl (K.T.); slawomir.kazmierski@cbmm.lodz.pl (S.K.); artur.rozanski@cbmm.lodz.pl (A.R.); marek.potrzebowski@cbmm.lodz.pl (M.P.); krystyna.rozga-wijas@cbmm.lodz.pl (K.R.-W.); 2 Department of Inorganic and Analytical Chemistry, Poznan University of Medical Sciences, Rokietnicka 3, 25-406 Poznan, Poland; lsobotta@ump.edu.pl; 3 Department of Ceramics and Refractories, Faculty of Materials Science and Ceramics, AGH University of Science and Technology, A. Mickiewicza 30, 30-059 Krakow, Poland; ziabka@agh.edu.pl; 4 Department of Medical Biology, Institute of Biology, Jan Kochanowski University, Swiętokrzyska 15, 25-406 Kielce, Poland; magdalena.plodowska@ujk.edu.pl; 5 Institute of Physics, Jan Kochanowski University, Swiętokrzyska 15, 25-406 Kielce, Poland; k.szary@ujk.edu.pl; 6 Holy Cross Cancer Center, S Artwinskiego 3, 25-734 Kielce, Poland

**Keywords:** hybrid yeast–silica capsules, encapsulation of phenosafranin and azure dyes, fluorescent biomimetic capsules

## Abstract

Yeast capsules (YCs) produced from *Saccharomyces cerevisiae* with encapsulated fluorescent phenosafranin and azure dyes were used as catalytic template guides for developing hybrid functional organic/inorganic hollow microstructures with silica (SiO_2_) deposited on their surface generated in the imidazole-buffered system without the addition of any cationic surfactant. YCs-doped with SiO_2_ act as fluorescence emitters maintaining dye-loaded materials by sealing the microporous surface of YCs. We used vinyltrimethoxysilane as a precursor of SiO_2_ endowed with functional vinyl groups facilitating their further modification without disturbing the polysaccharide wall integrity. Consequently, the hybrid fluorescent polysaccharide/silica microcapsules (YC@dye@SiO_2_) are promising for wide-ranging optoelectronic applications in electrochromic and OLED devices with biocompatibility and biodegradability properties.

## 1. Introduction

Recently, significant focus has been directed towards organic–inorganic hybrid materials, leveraging the advantages of both components by incorporating inorganic molecules into naturally occurring organic templates like yeasts. Unicellular organisms, such as diatoms (microalgae) [1], radiolaria, synurophytes, and multicellular sponges are encased with silica. Cell silicification technology has great potential to improve biological protection against microbial attacks. It enables the development of new classes of biomimetic materials for use as biocatalysts, and cellular sensors in cell therapy, probiotic delivery, and artificial cell development [2,3,4]. Silica-based materials have gained significant interest in biomedical and biotechnological fields [5]. The silicification of single native cells observed in nature encourages researchers to cover yeast cells or living organisms with silica coatings to enhance their stability and provide new functional cellular properties or advanced yeast–silica biohybrid hollow microstructures [6,7]. Hence, biomimetic silicification is conducted in the aqueous medium in the presence of cationic polyelectrolytes such as polyamines under mild conditions (i.e., room temperature, ambient pressure, and near-neutral pH values) [8,9,10,11]. Polyamines endowed with quaternary amine groups are known to affect silica formation by catalyzing siloxane-bond generation and acting as flocculating agents [12]. Thus, the silicification process was used to form a mechanically durable silica coat on the surface of individual cells under physiologically mild conditions [13,14]. Various strategies were developed [15] to compose the silica shell, which is easily achieved via silane coupling chemistries involving the surface silanol groups. Several cationic polyamines [16,17,18,19,20,21] have been used as catalytic templates for silica condensation, e.g., natural or synthetic polymers including peptides such as SurSi [22], RSGH [23], R4C12R4 [24], poly-L-arginine [25,26], poly-L-lysine [27], poly(allylamine hydrochloride) [28], modified chitosan [29], poly(diallyldimethylammonium chloride) [30,31,32,33,34,35], polyethyleneimine [36,37], amine-terminated dendrimers structures [38,39], and copolymers from polybutadiene-*co*-poly(2-vinylpyridine) [40]. Polyamines incorporated in the silica shell deliver a positive charge on the surface and play critical roles in the silica nucleation and the continuing growth of the silica shell at the interface via a sol–gel process. Electrostatic interactions of cationic species with the yeast cell surface are a key factor in directed mineralization containing regions rich in charged functional groups [41,42]. The capsular polysaccharides with the net negative charge on their surface can serve as hosts for cationic, hydrophilic dye molecules entrapped within their walls. In our research, we used phenazine and phenothiazine dyes exemplified by phenosafranin (PSF) and azure A (AZ), respectively, containing the electron-donating amine group. These dyes provide numerous binding sites for adsorption onto anionic polysaccharide capsule walls endowed with hydroxyl functional moieties. The ‘host–guest’ interactions between these components promote the electrostatic attraction for other reagents such as silica oligomeric species according to the layer-by-layer (LbL) method. Reactive two-amine groups of PSF provide abundant binding sites for adsorption and cross-linking reactions between the polysaccharide capsule shell and SiO_2_ endowed with functional vinyl groups. Thus, silica entrapped within biological matrices can improve their physical, thermal, and mechanical properties. The high biodegradability and low cytotoxicity of silica make such biohybrid structures ideal as probes for medical purposes. Moreover, the sol–gel process of silicification facilitates the incorporation of colorants and other elements, along with introduced functional groups, into the silica shell through entrapment. This approach engineered capsules with polysaccharide wall-reinforced SiO_2_ with incorporated photosensitive PSF and AZ dye molecules (YC@dye@SiO_2_) to form fluorescence emitters with a glass envelope. Numerous examples in the literature demonstrate how silicification can help encapsulate fragile biomolecules (i.e., enzymes, stem cells, proteins) [43,44,45] to obtain biocatalysts and biosensors covered with a silica shell. Still, only a few articles have described the encapsulation of dyes within cells functionalized using a silica coating [46]. In this study, we used YC@PSF and YC@AZ as catalytic templates for biomimetic silicification and structure-directing agents for SiO_2_ obtained in the imidazole-buffered system without the introduction of cationic quaternary ammonium salt such as a commonly used cetyltrimethylammonium bromide (CTAB) [47]. We report a simple and versatile approach to introduce chemical functional groups to biomimetic silica structures, to apply them to yeast capsules, which can be a promising material in optical chemistry. This innovative strategy aims to provide new insight into developing safe imaging probes that mimic nature. Moreover, such biocompatible YC@dye@SiO_2_ capsules with entrapped PSF and AZ dyes may have great applications in the industry in electrochromic devices, solar energy cells, optical sensors, and photovoltaic cells.

## 2. Results and Discussion

**Figure 1:**
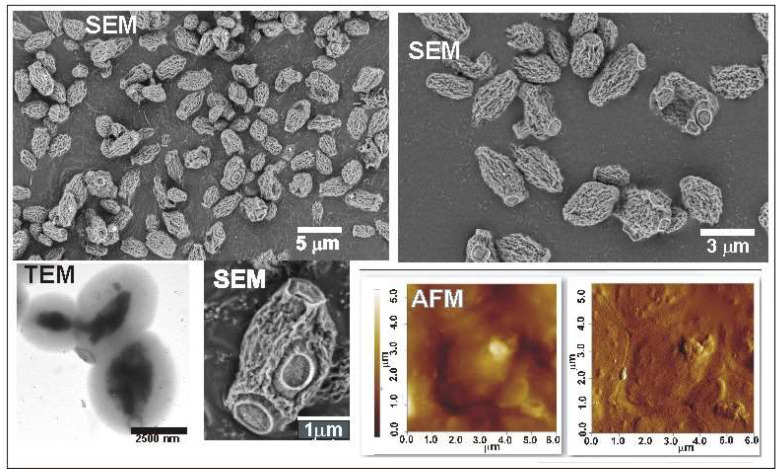
Representative micrographs of lyophilized YCs were captured using a scanning electron microscope (SEM). The transmission electron microscope (TEM) indicates empty capsules. Atomic force microscopy (AFM) provides a topographic image and phase contrast of a YC at the same location.

**Figure 2:**
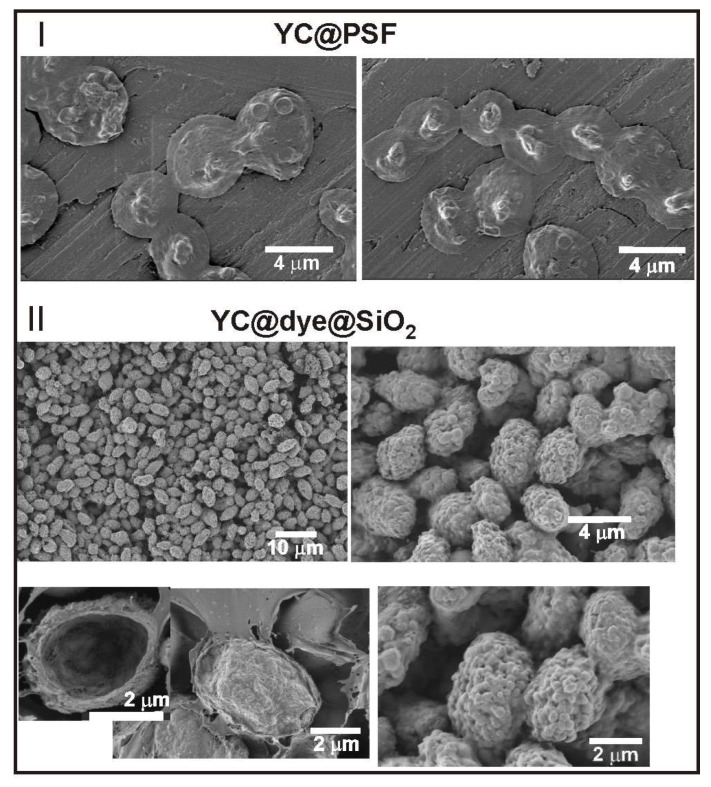
Panel (**I**) illustrates SEM micrographs of YC@PSF loaded with a dye before modification. Panel (**II**) shows YC@PSF@SiO_2_, microcapsules after in situ polymerization of the VTMS precursor, and roughness results from the coating, with visible silica aggregates.

The outermost layer of polysaccharides surrounding capsules comprises β(1,3)(1,6)-glucan chains that physically interact and tether microfibrils. The branched microfibrils form inter-polymer hydrogen bonds via their hydroxyl groups [48,49]. The yeast capsules (YCs), characterized by their porous surface and net negative charge, can form strong electrostatic interactions with positively charged PSF and AZ dye molecules facilitating their entrapment. The LBL process was initiated with dyes containing amine groups through electrostatic interactions with hydroxyl groups on the YCs’ surface and concurrently finished with dyes that enhanced catalytic interactions with silicic acid derivatives of VTMS at the outer interface, thereby accelerating the formation of SiO_2_. According to the LBL method, these interactions are essential for the targeted deposition of silica on the YC wall [50,51]. The surface morphology of native YCs and modified YC@dye@SiO_2_ characterized by SEM, TEM, and AFM techniques are shown in (Figure 1) and (Figure 2), respectively. Imidazole in the PBS buffer might act as an external catalytic pair (general base) of PSF and AZ dyes in the silicification processes under mild conditions described by Morse et al. [52]. Our approach differs from conventional methods because it relies on the growth of hydrophobic SiO_2_ selectively on the polysaccharide YC surface endowed with phenazine dyes with functional amine groups. The synthesis involved in situ silicification of a hydrophobic VTMS on a positively charged layer with deposited amine cationic dyes producing YC@dye@SiO_2_ microcapsules decorated with functional vinyl groups.

**Scheme 1:**
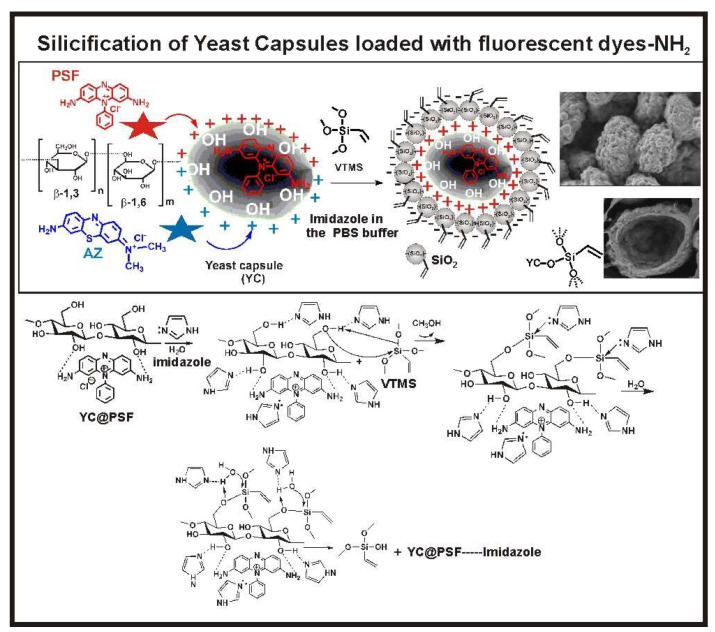
Diagram of the synthesis of fluorescent capsules reinforced with a SiO_2_ coating. The hydroxyl groups in β(1,3)-glucan chains are involved in hydrogen bonds with imidazole that can catalyze reactions with VTMS. The proposed mechanism is based on forming a transient pentavalent silicon species, stabilized by a donor bond to the nitrogen of the imidazole ring suggested by Morse [52].

Moreover, this process was conducted without adding any cationic surfactant acting as a structure-directing agent (e.g., CTAB). Thus, polysaccharide capsules with entrapped dyes YC@PSF and YC@AZ fulfilled the role of guides and cooperative agents for the self-assembly of SiO_2_ from the VTMS alkoxysilane precursor, controlling silica nucleation and growth. Previously, capsules with an inorganic layer on the YC surface, as reported by Weinzierl [53], were prepared via the condensation of silanol groups during the sol–gel process. This procedure used tetraethyl orthosilicate (TEOS) in water–alcohol mixtures as the solvent, with ammonia, according to the Stöber method, which requires harsh conditions (extremes of pH > 10, elevated temperatures above 80 °C). In contrast, our biomimetic procedure consists of simple positively charged PSF or AZ adsorbed onto the YC surface, where imidazole catalyzes the polycondensation of silicic acids to form silica under ambient conditions (at neutral pH and low temperature). Imidazole moieties, also acting as counteranions, significantly affect silica morphogenesis on YC surfaces by interacting with dyes and modulating charge density on the YC@dye outer shell (Scheme 1). The catalytic mechanism involves the dual features of the imidazole group–its ability to form hydrogen bonds with silicic acid and electrostatic attraction towards oligomeric silicic acid species [54]. The nucleophilic attack by -OH groups is essential for the hydrolysis VTMS precursor, while the hydrogen-bond acceptor groups, such as -NH_2_, facilitate the hydrolysis reaction. We presume, that amine groups of dyes can act as a general acid-base catalyst where the deprotonated amine groups (“base”) abstract a proton from the silicic acid derivative, leading to the formation of the reactive silanolate, and protonated amine groups (“acid”) facilitate the release of water by the protonation of silicic acid [55]. Yang et al. [56] used tetramethyl orthosilicate (TMOS) with (3-Mercaptopropyl)trimethoxysilane (MPTMS) to cover yeast cells with a silica shell containing the thiol functional groups (SiO_2_^SH^) for specific reactions with maleimide derivatives under biocompatible conditions. Kamanina et al. [57] applied diethoxydimethylsilane (DEDMS) and TEOS as precursors to form an organosilica coating around microorganisms to develop a biochemical oxygen demand biosensor. Thus, one significant benefit of silica polymers is the formation of porous inorganic coatings that are optically transparent, known as sol–gel glass. Therefore, we produced fluorescent hybrid inorganic/organic replicas of YCs with entrapped dyes, functionalized with silica-containing vinyl groups on their surface, designed as YC@PSF@SiO_2_ and YC@AZ@SiO_2_ with fluorescent properties. Moreover, the yeast-based β-glucan system has demonstrated an effective strategy for oral drug delivery of various therapeutics [58]. Therefore, YC@dye@SiO_2_ can aid in oral administration, helping to overcome the biological barriers of the harsh gastrointestinal environment. Moreover, reactive vinyl moieties on SiO_2_ allow YCs to remain accessible for interaction with external species in their environment. By binding biotinylated compounds, they can achieve targeted therapeutic effects by specifically recognizing immune cells [59].

### 2.1. Characteristics of YCs and YC@dye@SiO_2_

YCs were prepared using the extraction method reported earlier [60]. They exhibited a typical ellipsoidal morphology with sizes ranging from 2.5 to 4.0 μm, as confirmed by images from AFM, SEM, and TEM (Figure 1). We obtained a uniform YC with an ellipsoidal shape and smooth surface. Thus, YCs serve as templates to guide the coating process with silica, producing accurate replicas (Figure 2).

The polysaccharide wall of YCs is resistant to acidic and basic conditions, or organic solvents, making it ideal for versatile silane technology. Our results demonstrate that the presence of cationic-charged molecules PSF and AZ dyes adsorbed on the surface using the LbL method then encapsulated by the passive diffusion process and modifying the YC wall facilitated the precipitation and deposition of SiO_2_ with the imidazole-buffered system in mild conditions. Dye molecules with amine groups adsorbed on the YC scaffold can work as a silane coupling agent and act as catalysts in a silicification process at near-neutral pH in aqueous solutions. Silicic acid molecules electrostatically interact with amine groups of dyes that were adsorbed on the YC surface and precipitated silica from an aqueous solution producing YC-doped SiO_2_. The SEM micrographs indicate morphological differences on the YC@dye@SiO_2_ surface (Figure 2) compared to the native YCs before modification (Figure 1). SEM images (Figure 2) show that VTMS can self-assemble to form well-defined silica nanoaggregates. Thus, YC@dye@SiO_2_ capsules were formed with inner diameters determined by the size of the YCs under physiologically mild conditions, inspired by bio-silicifications. The YC covered with SiO_2_ was slightly larger and adhered nanostructures were uniformly distributed on its surface. Consequently, the synthesis of the YC@dye@SiO_2_ microcapsules using hydrophobic silica precursors in the reaction of in situ polymerization by condensation showed the forming of SiO_2_ binding to the outer surface of the YCs. Polymerization of VTMS preferably occurs on the YC surface thus, micropores of naked YCs were sealed by silica (Figure 2).

**Figure 3:**
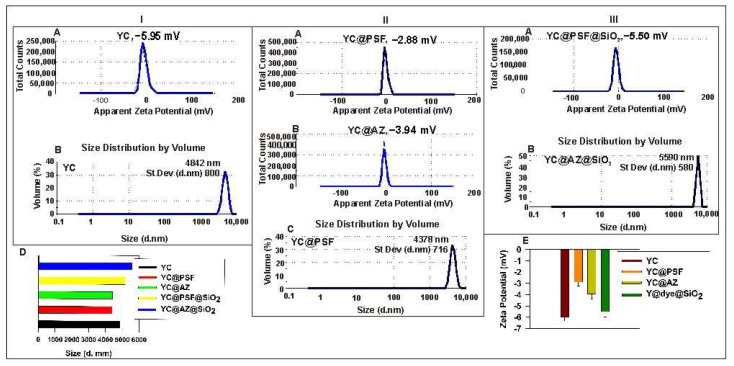
Panel (**I**) depicts the zeta potential (**A**) and size distribution (**B**) of the parent YCs. Panel (**II**) presents the zeta potential results of YC@PSF (**A**) and YC@AZ (**B**), with a representative size distribution of YC@PSF shown in (**C**). Panel (**III**) displays zeta potential data from electrophoretic mobility measurements for YC@PSF@SiO_2_ (**A**) and YC@AZ@SiO_2_ (**B**). Diagrams (**D**,**E**) provide a comparative overview of all samples’ size distribution and zeta potential.

Therefore, electrostatic interactions of charged and polarized groups of YCs polysaccharide surfaces covered with cationic PSF or AZ amine dyes led to oversaturation and may explain heterogeneous nucleation of the silica acid molecules from solution and selective growth of silica on the YCs’ surface. SEM micrographs of the YC@dye@SiO_2_ samples showed that the silica was well-dispersed on the YC surface after modification using a hydrophobic VTMS precursor. The SiO_2_ primarily precipitated on the YC surface and remained attached after purification. Consequently, the resulting YC@dye@SiO_2_ microcapsules covered with silica were stable. The zeta potential analyses were applied to evaluate the surface characteristics of YC@PSF, and YC@AZ capsules before and after silica modification (Figure 3). The YCs initially possessed a negative charge of approximately −5.95 mV due to their primary composition of β(1,3)-glucan. The zeta potential altered to be −2.88 nm and −3.94 mV with the deposited cationic PSF and AZ dye molecules, respectively (Figure 3). It suggests electrostatic interactions between the positively charged dyes and the negatively charged regions of the YCs. The data confirm that the dyes were adsorbed onto the YCs successfully. Moreover, the fabricated coating from SiO_2_ on the YC@dye surface was evidenced by a decrease in zeta potential to −5.50 mV after the silicification.

### 2.2. Chemical Composition of YC and YC@dye@SiO_2_

#### 2.2.1. ^13^C CP MAS NMR and ^29^Si CP MAS NMR Analyses of YC@dye@SiO_2_ Microcapsules in Solid State

**Figure 4:**
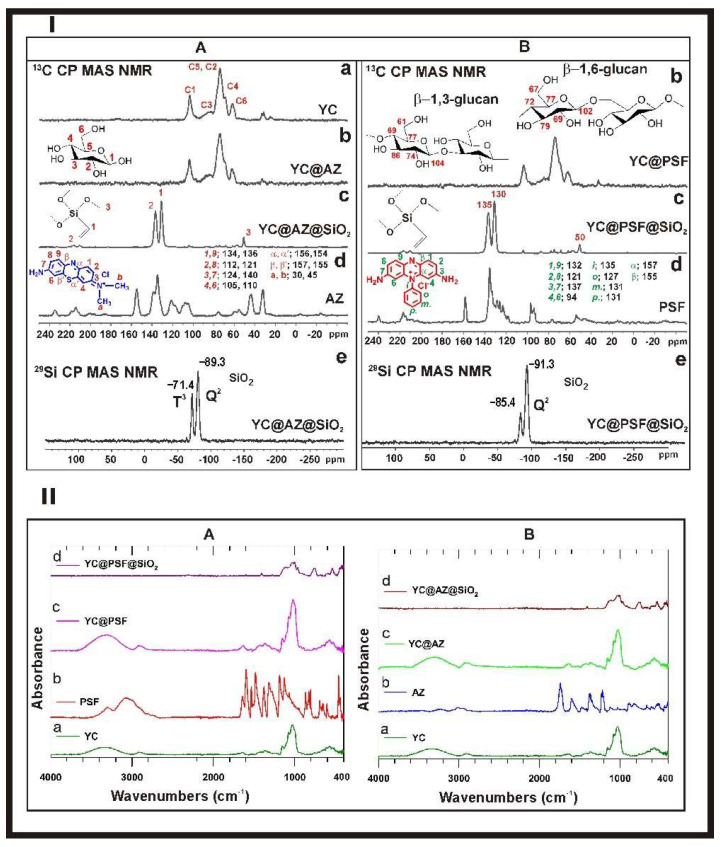
Panel (**I**): ^13^C CP MAS NMR and ^29^Si CP MAS NMR spectra showing: (**A**) YC, PSF, YC@PSF, and YC@PSF@SiO_2_. (**B**) YC, AZ, YC@AZ, and YC@AZ@SiO_2_. Panel (**II**): Representative FT-IR spectra (4000–400 cm^−1^) illustrating: (**A**) YC (a), PSF (b), YC@PSF (c), and YC@PSF@SiO_2_ (d). (**B**) YC (a), AZ (b), YC@AZ (c), and YC@AZ@SiO_2_ (d).

The ^13^C NMR assignments of YC, YC@dye, YC@AZ@SiO_2_, and YC@PSF@SiO_2_ approved the encapsulation of dyes and structure of capsules from β(1,3)(1,6)-glucans [61,62,63,64] (Figure 4I). Moreover, ^29^Si NMR solid-state spectra of YC@dye@SiO_2_ showed that silica covered the YCs surface.

Thus, the polysaccharide surface modification was successfully performed in the imidazole-buffered system using VTMS as a monomer to generate SiO_2_ with functional vinyl groups in mild conditions. Silica was formed in situ by the sol–gel approach and the YC surface was engineered by the adsorption of phenazine and phenothiazine dyes containing amine groups. ^29^Si SS-NMR spectra (Figure 4I(Ae)) and (Figure 4I(Be)) showed typical signals assignable to a Si-O bond: signals at −85.4 ppm, −89.3 ppm, and −91.3 ppm correspond to Q^2^ shifts [65]. The presence of Q^2^ species indicates an incomplete condensation process, i.e., silanol groups (Si-OH) or alkoxy groups can be found in the network. The signal at −71.4 ppm described T^3^ structures indicating a high condensation degree of a polysiloxane network.

#### 2.2.2. FT-IR Analyses of YC@dye and YC@dye@SiO_2_

The representative FT-IR spectrum of YCs illustrates the vibrational absorption of the polysaccharide backbone at 1160 and 1153 cm^−1^ for ν(C-O-C, and CC) and the stretching at 1041 cm^−1^ for ν(C-O) (Figure 4II, a) [66]. The YC spectrum showed bands at 1108 cm^−1^ attributed to ν(C-O) polysaccharide stretching. The bands at 3500–3000 cm^−1^ and 3000–2800 cm^−1^ are characteristic of ν(C-OH) and ν(CH_2_, CH) stretching, respectively, typical of polysaccharides. The asymmetric and symmetric stretching of (-COO) groups of YC is observed at small bands at 1640 cm^−1^ and 1408 cm^−1^. Hydrogen bond formation between the YC polysaccharide wall, amine groups of PSF or AZ, and hydroxyl groups of silicon nanoparticles likely contributed to the region (1200–900 cm^−1^). Furthermore, the fingerprint region of AZ [67] and PSF, with signals at 868, 827, 806 cm^−1^; 694, 654, 607 cm^−1^; and 462 cm^−1^ [68,69], assigned to aromatic ring torsion in-plane modes, was also evident in the YC@PSF@SiO_2_ spectra. Hybrid inorganic/organic capsules coated with SiO_2_NPs containing a functional vinyl group showed vibrational absorption at 1650 cm^−1^, 1380 cm^−1^, 1190 cm^−1^, 1100–990 cm^−1^, 990 cm^−1^, and 930 cm^−1^, as well as 750 cm^−1^. The silicon structural fingerprint vibrations region characteristic of SiO_2_ was located at infrared bands in 1130–1000 cm^−1^ associated with the sequential stretching Si-O mode from the (Si-O-Si) network [70]. The Si-O-Si peak at 1042 cm^−1^ shows that VTMS was successfully adsorbed on the YC surface. The symmetric mode of the band corresponding to the (Si-O-Si) vibrations was also detected at 790 cm^−1^, and ν(Si-O(H)) stretching at 950 cm^−1^. The (Si-O) rocking mode was observed at 540 cm^−1^. The absorption band in 1380 cm^−1^ and 1190 cm^−1^ regions corresponds to the bending vibrations of the (Si-CH=CH_2_) unreacted vinyl groups of silane which are not subjected to hydrolysis.

### 2.3. Physical Characteristics of Capsules Using TGA and Nitrogen Adsorption/Desorption Analyses

**Figure 5:**
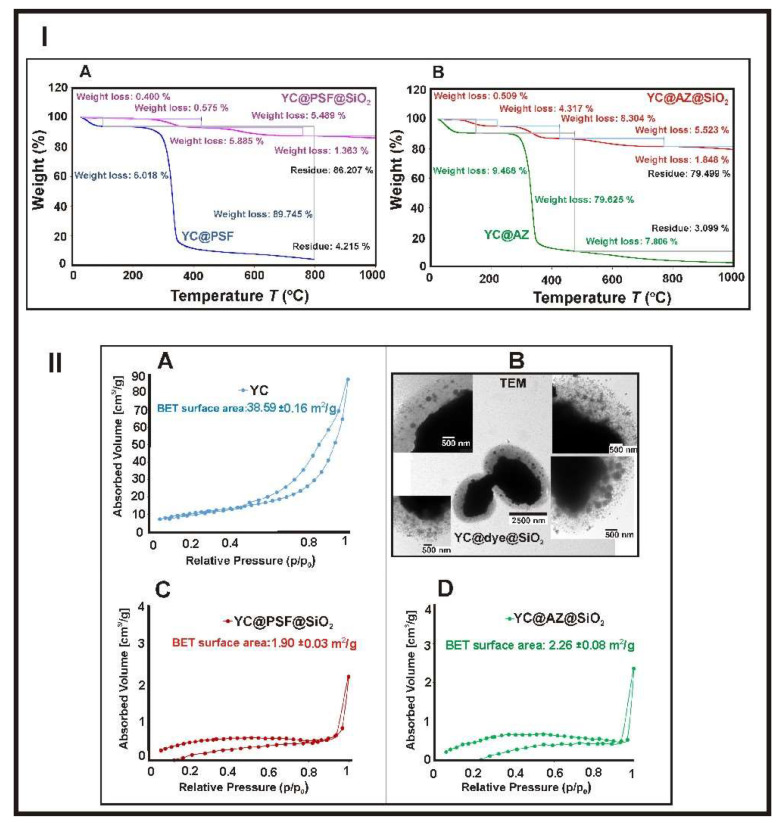
Panel (**I**): TGA samples in the sequence: (**A**) YC@PSF and YC@PSF@SiO_2_, (**B**) YC@AZ and YC@AZ@SiO_2_. Panel (**II**): Nitrogen adsorption–desorption isotherm of YC (**A**), YC@PSF@SiO_2_ (**C**), and YC@AZ@SiO_2_ (**D**). The TEM micrograph of YC@dye@SiO_2_ shows sealed YC’s macropores by SiO_2_ (**B**).

Thermogravimetric analysis (TGA) was performed to confirm the successful synthesis of YC@dye@SiO_2_ microcapsules. It can be seen from TGA profiles (Figure 5I), that the first stage (20–100 °C) of mass loss of YC@PSF and YC@AZ (5.5%) was attributed to the desorption and removal of physically adsorbed water. The decomposition of hybrid YC@dye@SiO_2_ microcapsules begins at slightly higher temperatures than YC@dye, in which SiO_2_NPs cause the blocking effect. The major weight loss of YC@dye (approximately 85–90%) in the second stage was located in the range of 100–350 °C was ascribed to the dehydration of some functional groups of saccharide rings (e.g., -OH, -COOH), and the breaking of C-O-C glycosidic bonds of YCs [71,72,73]. The third stage (400–1000 °C) of mass loss (10%) of YC@dye was assigned to the intermolecular crosslinking of oligosaccharides by dehydration at higher temperatures. In contrast, multiple degradation profiles of YC@PSF@SiO_2_, and YC@AZ@SiO_2_ can be divided into four stages and the last step (700–1000 °C) revealed the residue mass of reported samples 86.2%, and 79.5%, respectively. This was attributed to the incomplete decomposition of SiO_2_ with the cross-linked (-Si-O-Si-) bonds deposited on the YC surface.

It confirmed excellent thermal stability greater than 800 °C of the hybrid YC@dye@SiO_2_ capsules. Thus, the advantage of using VTMS was to provide additional thermal stability to YCs forming an inorganic silica shield. The YC@dye@SiO_2_ microcapsules were successfully synthesized.

Nitrogen adsorption–desorption N_2_ isotherms for the parent YCs and YC@dye@SiO_2_ showed a type H3 hysteresis [74] (Figure 5II). Specific surface areas were calculated using the standard Brunauer–Emmett–Teller (BET) method. The result of BET surface area measurements for YCs was 38,59 m^2^/g, which decreased to 1.90 m^2^/g and 2.2 m^2^/g for YC@PSF@SiO_2_ and Y@AZ@SiO_2_, respectively. Thus, a reduction in BET surface area, accompanied by a proportional decrease in pore volume, indicates the successful surface modification of YCs by SiO_2_. Therefore, YCs were transformed into a high value-added material, YC@dye@SiO_2_ microcapsules, with multifunctional properties due to functional vinyl groups, which are useful for further reactions. Undoubtedly, the inorganic SiO_2_ shield successfully closed off the pores of the YCs. The silica effectively sealed the pores on the YC surface, preventing the release of dyes. Thus, the pores of YCs were capped with the pore-encapsulated silica guest.

### 2.4. Spectrophotometric and Fluorescence Analyses of YC@dye@SiO_2_ Microcapsules

**Figure 6:**
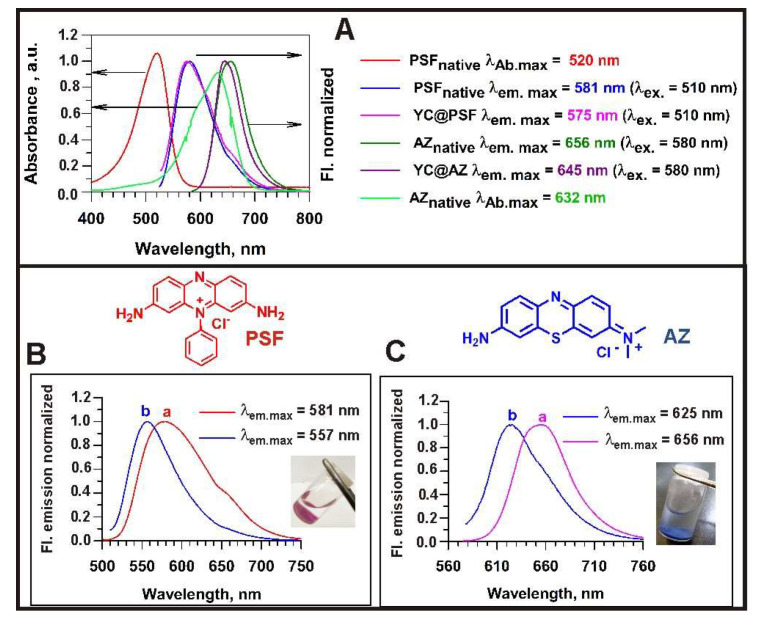
(**A**) shows the absorption spectra of parent PSF and AZ dyes dissolved in water, along with their normalized fluorescence emission spectra: PSF (λ_ex_ = 510 nm, λ_em.max_. = 581 nm) and AZ (λ_ex_ = 560 nm, λ_em.max_. = 655 nm). Entrapped dyes, YC@PSF (λ_ex_ = 510 nm, λ_em. max._ = 575 nm) and YC@AZ (λ_ex_ = 560 nm, λ_em.max._ = 645 nm), exhibited blue shifts of 6 nm and 11 nm, respectively. (**B**) and (**C**) present the normalized fluorescence emission spectra of YC@PSF@SiO_2_ and YC@AZ@SiO_2_, respectively. The two inset photos represent purified YC@AZ and YC@PSF samples sedimented in water with incorporated dyes.

Cationic amine dyes were easily captured on the polysaccharide surface of YCs through electrostatic forces and then entrapped via passive diffusion across the semipermeable shell and water-swollen pores. The adsorbed, PSF and AZ molecules permeated the YCs forming strong molecular complexes at a polar site by electrostatic interactions and intermolecular hydrogen bonds between their primary amine groups and hydroxyl groups of the β(1,3)-glucan chains. Consequently, the YCs acted as electron donors, while the cationic dye molecules served as electron acceptors. The UV–Vis absorption and fluorescent spectra of native PSF and AZ dyes in water solutions and encapsulated within YCs (YC@PSF, YC@AZ) are illustrated in (Figure 6A). PSF excited at a wavelength of λ_ex_ = 510 nm, showed the maximum emission at λ_em.max_. = 581 nm for the native and 575 nm for the entrapped dye. AZ was excited at a wavelength of λ_ex_ = 560 nm, showing the maximum emission at λ_em.max_. = 655 nm, for the parent and 645 nm for the incorporated dye.

**Figure 7:**
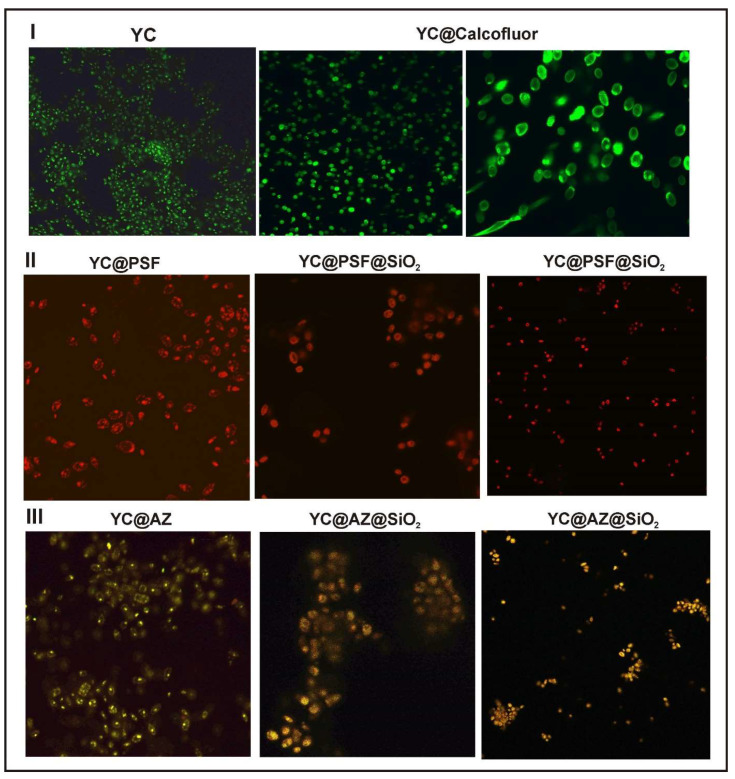
Images of microcapsules captured from a confocal fluorescent microscope using FITC (λ_ex_ = 467–498 nm, λ_em_ = 513–556 nm) and Texas Red (λ_ex_ = 559 nm, λ_em_ = 630 nm ± 34 nm) filters. Panel (**I**): YC and YC stained calcofluor, applied as a standard marker of polysaccharides. Panel (**II**): images of YC@PSF and YC@PSF@SiO_2_ obtained using the FITC filter. Panel (**III**): images of YC@AZ and YC@AZ@SiO_2_ captured using the Texas Red filter.

Thus, both dyes entrapped in YCs exhibited a blue shift to a shorter wavelength of higher energy in fluorescence spectra. The hypsochromic shift of PSF and AZ was 6 nm and 10 nm, respectively. This proves electrostatic attraction between the cationic fluorophore and the anionic moieties of β(1,3)-glucan chains of YCs. The blue shift indicated a decreasing polarity in the environment of the dyes, which were entrapped by polysaccharides compared to bulk water. After modification of the YCs labeled with dyes involved in depositing VTMS precursor in situ polymerized, both dyes remained within YCs. This suggests that the SiO_2_ sealed the pores of the YCs polysaccharide network. The fluorescence emission spectrum of YC@PSF@SiO_2_ suspended in water, when excited at a wavelength of λ_ex_ = 490 nm, showed the maximum emission at λ_em.max_ = 557 nm (Figure 6B, curve b). Thus, entrapped PSF exhibited a blue shift of 22 nm (a hypsochromic shift) to a shorter wavelength of higher energy. The PSF dye endowed with amine groups is a dual-functional reagent that can cross-link polysaccharide YCs and SiO_2_, forming block-like irregulate clusters between them. Similarly, excitation of AZ at λ_ex_ = 570 nm revealed maximum emission at λ_em.max_ = 625 nm for encapsulated YC@AZ@SiO_2_ showing a blue shift of 30 nm (Figure 6C, curve b). The changes in the emission maximum compared to native dyes may exhibit that dyes have restricted intermolecular rotation or covalent bonding of dyes to YCs due to the synthesis of SiO_2_ [75]. Moreover, this blue shift indicates that the captured phenazine [76] and phenothiazine [77] dyes were embedded within the polymer network. In addition, a blue shift is attributed to restricting the rotation of the amine groups of the dyes, which become involved in intramolecular charge transfer [78]. Furthermore, the entrapped cationic dyes did not change color, suggesting the formation of ion pairs associated with dye-β(1,3)-glucan salt formation. Further investigation of the fluorescent properties of YC@dye@SiO_2_ using a confocal fluorescence microscope confirmed the entrapment of dye molecules within the hybrid microcapsules (Figure 7).

### 2.5. Optical Microscopic Fluorescence Analysis of YC@dye@SiO_2_ Microcapsules

The YCs and SiO_2_ in particular are almost transparent; therefore, hybrid hollow structures made from these materials are expected to be ideal for entrapped dye molecules. YCs can easily adsorb PSF and AZ with fluorescent properties constituting valuable templates for accurately adjusting the shape and size of SiO_2_ deposited on their surface. Moreover, silica protects the entrapped fluorophores within YCs from harmful species in the surrounding environment. The resulting YC@PSF and YC@PSF@SiO_2_ were observed under an optical fluorescent confocal microscope using the FITC (λ_ex_ = 467–498 nm, λ_em_= 513–556 nm) filter. Images of YC@AZ and YC@AZ@SiO_2_ microcapsules were registered using the Texas Red (λ_ex_ = 559 nm, λ_em_ = 630 nm ± 34 nm) filter, as illustrated in (Figure 6). The images revealed that PSF and AZ dye molecules were mainly entrapped within the YC wall and bound through electrostatic interactions with hydroxyl groups derived from polysaccharides or silica. Consequently, the silica enriched with negatively charged SiO^-^ groups interact through electrostatic forces and additional hydrogen bonds with cationic dyes which can aggregate with SiO_2_. Moreover, the pores of YCs were capped with the pore-encapsulated guest SiO_2_. This unique feature generates the system with “no-leaking” capability, even in the case of incomplete capping, meaning that the individual YC pores can still serve as independent reservoirs for encapsulating other molecules.

To sum up, a novel method of silicification in the imidazole-buffered system of the YC@dye using a hydrophobic VTMS precursor was established to obtain stable fluorescent microcapsules. The pores of the YCs were sealed by SiO_2_, preventing the release of the entrapped dye molecules. The synergistic approach is expected to facilitate the successful delivery of imaging probes, leveraging the binding capacity of dyes to design new biohybrid microcarriers that can work as fluorescent emitters.

### 2.6. Analysis of Singlet Oxygen Generation by PSF and YC@PSF@SiO_2_

Phenosafranin demonstrated a high singlet oxygen quantum yield in DMSO (Φ_Δ_ = 0.63) and moderate yield in DMF (Φ_Δ_ = 0.23), as shown in Table 1, compared to the standard ZnPc, which had quantum yields of Φ_Δ_ = 0.56 in DMF and 0.67 in DMSO.

**Figure 8:**
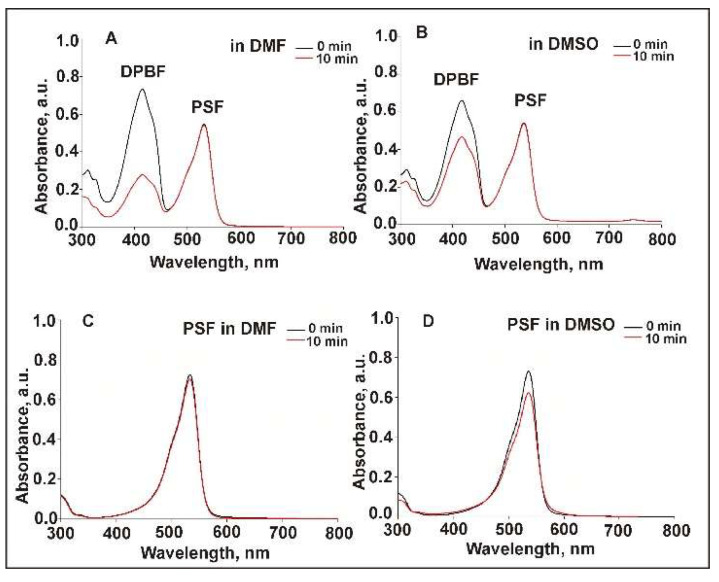
Spectra of PSF and DPBF dissolved in DMF (**A**) and DMSO (**B**), before and after visible light irradiation. Spectra of native PSF dissolved in DMF and DMSO (**C** and **D**, respectively), show the changes before and after visible light irradiation.

Conversely, none of the presented PSF formulations (i.e., YC@PSF, YC@PSF@SiO_2_) showed significant singlet oxygen generation, possibly due to the limited accessibility of molecular oxygen and light to the photosensitizer caused by the carrier’s armor. The native dye exhibited good photostability in both solvents, with quantum yields calculated to be near 10^−6^ (Table 1); however, its stability in DMSO was lower than in DMF. The results are consistent with the literature [79], which reports that hydrogen bonding significantly contributes to stabilizing the PSF cation in solvents. In a protic medium such as DMF, PSF, with the two amine groups and one positively charged nitrogen atom in the phenazine ring can form hydrogen bonds with DMF, thereby enhancing solubility as the dye molecule’s dipole moment increases. Although PSF had lower photostability than the reference ZnPc, its singlet oxygen generation did not increase (Figure 8). The photophysical behavior of entrapped phenazine dyes changes depending on the ground state aggregation. It decreases the fluorescence intensity and the formation of singlet oxygen (^1^O_2_) due to electrostatic interactions and hydrogen bonds with a polysaccharide YC wall and SiO_2_. Moreover, the nature of aggregates formed in different negative interfaces, specifically silica, is also important. Thus, aggregation and medium composition effects changed the energy transfer process among triplet states species and molecular oxygen. Moreover, polysaccharides were reported to have scavenging effects on superoxide and hydroxyl radicals and have antioxidant power reducing oxidative stress [80].

## 3. Materials and Methods

### 3.1. Materials

Commercial baker’s yeast (*Saccharomyces cerevisiae*) was purchased from a local grocery store. Vinyltrimethoxysilane H_2_C = CH-Si(OCH_3_)_3_ (VTMS, purity > 99%) was used as received from ABCR Company (Karlsruhe, Germany). Phenosafranin (3,7-Diamino-5-phenylphenazinum chloride, PSF, dye content ≥ 80%), and Azure A (3-amino-7-(dimethylamino)phenothiazine-5-ium chloride, AZ, dye content ≥ 70%) were purchased from Merck (Darmstadt, Germany). All other chemicals and organic solvents, such as imidazole, isopropanol, and acetone, used in this work were of reagent-grade quality, obtained from POCH (Gliwice, Poland), and used as received without further purification.

### 3.2. Synthesis

#### 3.2.1. Preparation of Inactivated Yeast Cells in Capsule Form

Yeast cell wall capsules (YCs) were prepared following the extraction method proposed by Soto et al. [81]. In short, 20 g of yeast (*Saccharomyces cerevisiae*) was suspended in 200 mL of 1 M NaOH solution to achieve a 10% (*w*/*v*) yeast suspension. In short, 20 g of yeast (*Saccharomyces cerevisiae*) was suspended in 200 mL of 1 M NaOH solution to achieve a 10% (*w*/*v*) yeast suspension. The suspension was heated to 80 °C and stirred for 1 h, then left to cool for 10–15 min with continuous stirring before being centrifuged at 2000× *g* for 10 min to pellet the cells. The supernatant was discarded, and the pellet was resuspended in 200 mL of distilled water, with the pH adjusted to 4.2. This suspension was heated to 55 °C for 1 h. The resulting sample was centrifuged at 2000× *g* for 10 min and thoroughly washed twice with distilled water. Finally, the YC wall pellet was washed four times with isopropanol, and twice with acetone. The resulting slurry was dried under vacuum at room temperature to obtain a white powder.

#### 3.2.2. Encapsulation of Dyes (PSF and AZ) Within YCs

Encapsulation of dyes within yeast capsules was achieved: 0.5 g of dry YCs obtained from *Saccharomyces cerevisiae* were mixed in a tube with 15 mL of distilled water, resulting in a uniform single YC suspension at room temperature (25 °C). Subsequently, a dye (33 mg) dissolved in 5 mL of distilled water, was added to the YC suspension and stirred in an orbital shaker for 12 h to facilitate dye loading. Capsules containing the entrapped dye (YC@PSF, YC@AZ), were then separated from the supernatant with excess colorant by centrifugation at 2000–1700× *g* for 3 min. The cell pellet fraction was resuspended in 15 mL of distilled water, and the washing step was repeated eight times to ensure purification. The resulting YC@PSF and YC@AZ were further processed using size-exclusion chromatography (SEC) by passing the suspension through a Sephadex G-50 gel (Merck, Darmstadt, Germany) filtration column, with distilled water as the mobile phase. The first color band containing YC@PSF or YC@AZ which moved faster, was separated from any traces of a parent dye and collected. The quantity of a dye encapsulated in YCs was determined spectrophotometrically at a wavelength of λ = 520 nm (for PSF) and λ = 630 (for AZ) using a calibration curve prepared for dye samples in water solutions within a linear response range. The estimated molar extinction coefficient of PSF (ε_520nm_ = 35,361 M^−1^ cm^−1^) and AZ (ε_630nm_ = 34,945 M^−1^ cm^−1^) were consistent with the literature values [82,83]. A comparison was conducted between the concentration of dyes in the initial solution before encapsulation and that in the removed supernatant. The YC@PSF and YC@AZ samples were sonicated in a methanol solution for 15 min and then centrifuged to release the encapsulated dye for measurement. The experiments revealed that 15 mg of PSF, and 36 mg of AZ were encapsulated per 1 g of YCs.

#### 3.2.3. Synthesis of YC@dye@SiO_2_ Microcapsules

The YCs containing encapsulated dyes (YC@dye) were used as a biological template in the precipitation reaction for silica deposition. The synthesis of the exoskeleton from silica creating a glass armor on the capsule shell was synthesized through the hydrolysis of vinyltrimethoxysilane (VTMS) in an imidazole-buffered solution (pH = 7.4). Capsules (0.1 g) with entrapped dyes YC@PSF or YC@AZ were dispersed in 5 mL of PBS (pH = 7.4) containing 0.08 g (1.18 × 10^−3^ M) imidazole. Then, a solution of 0.50 mL (3.27 × 10^−3^ M) VTMS was added. The suspension was gently mixed overnight on an orbital shaker to facilitate the gelation at room temperature. Finally, the silica-coated YC@dye@SiO_2_ product was collected by centrifugation at 3000× *g*, washed with distilled water, and purified by repeated centrifugation. After freeze-drying, the yield was 85%. Silica deposited on the YC@dye surface was confirmed using TGA, FTIR, and NMR analyses.

### 3.3. Microscopic (AFM, SEM, TEM) Analyses of the Surface Morphology of YC and YC@dye@SiO_2_ Microcapsules

The morphology of the YCs before and after coating by silica was characterized using scanning electron microscopy (SEM, ThermoFisher Scientific Apreo, Waltham, MA, USA). Transmission electron microscopy (TEM) micrographs were prepared using the TESLA BS500 instrument (Austin, TX, USA). Atomic force microscopy (AFM) images were captured using Park NX10 (Park Systems, Technolutions, Santa Clara, CA, USA). Images were prepared with the NCR-10 (40 N/m^2^) probe using the NCM technique.

### 3.4. Microscopic Fluorescence Analysis of YC@dye@SiO_2_ Microcapsules

The images of YC@dye and YC@dye@SiO_2_ were obtained from confocal microscopy (Leica TCS SP8, Mannheim, Germany). The observations were conducted using phase contrast microscopy and fluorescence imaging filters for FITC (excitation wavelength λ_ex_ = 467–498 nm, emission wavelength λ_em_= 513–556 nm) and Texas Red (λ_ex_ = 559 nm, λ_em_ = 630 nm ± 34 nm).

### 3.5. Fourier Transform Infrared (FT-IR) Measurements

FT-IR measurements were conducted using a Thermo Scientific Nicolet 6700 FT-IR instrument (Thermo Fisher Scientific, Waltham, MA, USA) equipped with a Golden Gate ATR, (Attenuated Total Reflectance) accessory with detector DTGS using a resolution of 2 cm^−1^ at a rate of 4 scans per second. The baseline was manually adjusted for all the spectra.

### 3.6. Thermogravimetric Analysis

Thermogravimetric analyses (TGA) of naked YCs and YC@dye@SiO_2_ were conducted under a nitrogen atmosphere. The analyses were performed with a heating rate of 10 K/min over a temperature range of 0–1000 °C, using a TGA 5500 apparatus from TA Instruments (New Castle, DE, USA).

### 3.7. Nitrogen Adsorption–Desorption Studies

The nitrogen adsorption–desorption isotherms were recorded at −196 °C using a Micromeritics ASAP 2020 Plus instrument (Norcross, GA, USA). Before measurements, samples were vacuum degassed using FloVac Degasser (Norcross, GA, USA) at 100 °C for 24 h. Specific surface areas were calculated using the standard Brunauer–Emmett–Teller (BET) method. A multi-point BET surface area was obtained from the nitrogen adsorption isotherm in the relative pressure range from 0.1 to 0.3.

### 3.8. Determination of Hydrodynamic Diameter and Zeta Potential of YCs, and YC@dye@SiO_2_ Microcapsules

The hydrodynamic diameters of naked YCs, YC@dye, and YC@dye@SiO_2_ were estimated using the dynamic light scattering (DLS) technique. Zeta potential measurements were obtained through electrophoretic mobility assays using a DTS1060C cell and a Zetasizer Nano-Z (Malvern Instrument, Malvern, UK).

### 3.9. Singlet Oxygen Quantum Yields Under Light Irradiation

Singlet oxygen quantum yields were determined in dimethyl sulfoxide (DMSO, Fisher, Hampton, NH, USA) and *N*,*N*-dimethylformamide (DMF, Fisher) at ambient temperature, following the comparative method described earlier [84,85,86]. This method utilizes 1,3-diphenylisobenzofuran (DPBF, Merck) as a chemical quencher of singlet oxygen, and unsubstituted zinc (II) phthalocyanine (ZnPc, Aldrich, St. Louis, MO, USA) as a reference compound. Mixtures of PSF with DPBF were irradiated in the Q-band region with light adjusted to the maximum absorbance wavelength, using the M250 monochromator (Optel, Opole, Poland). Changes in absorption spectra were recorded using an Ocean Optics Flame spectrophotometer (Orlando, FL, USA) equipped with a DT-MINI-2-GS light source (Orlando, FL, USA). Singlet oxygen quantum yields were then calculated using the previously presented equation [87,88].

Photodecomposition quantum yields. Photostability measurements were conducted in DMF and DMSO at ambient temperature under aerobic conditions, using the method earlier mentioned. A 150 W xenon lamp (Optel, Opole, Poland) was applied as the visible light source (>450 nm) isolated by an HCC-16 cut-off filter (Optel, Opole, Poland). Spectral changes during irradiation were recorded with an OceanOptics Flame spectrophotometer equipped with a DT-MINI-2-GS light source, and the photodegradation quantum yield was calculated.

## 4. Conclusions

We used phenazine and phenothiazine dyes, which were entrapped electrostatically onto the YC surface, and catalyzed polycondensation of silicic acid on the polysaccharide wall in the imidazole-buffered solution. We obtained fluorescent hybrid YC@dye@SiO_2_ hollow microstructures with a stable and rigid framework. The biomimetic microcapsule with vinyl-modified supported reagent VTMS can develop a system for fluorescence enhancement. The new fluorescent material was optically stable in organic solvents, and it can be used for further modification by addition via the C=C bond. YCs enveloped with silica endowed with vinyl moieties can be functionalized by thiol derivatives (RS-H) using coupling reactions for further applications of biologically active compounds or copolymerized with different vinyl monomers under UV light irradiation. This approach offers several advantages over conventional chemical methods because mixtures of alkoxysilane precursors can spontaneously react during the co-condensation process allowing for direct incorporation of functional groups (-NH_2_, -COOH) without additional chemical treatments. Consequently, the surface chemistry of YC@dye@SiO_2_ can be finely tuned for further specific applications. We believe, that various YC@dye@SiO_2_ microcapsules with chemical functionalities produced by the simultaneous addition of silane derivatives with reactive moieties will have great importance in optoelectronics fields. Moreover, obtained YC@dye@SiO_2_ are characterized by low aggregation, which positively influences their application value in optical technology. The new fluorescent biomaterial can serve as a matrix for porous fluorescent OLED biomembranes with long-term stability, as fillers, or as coating pigments, e.g., in the paper industry. The hybrid capsules are biocompatible, safe, and do not generate reactive oxygen species or harmful residues in the biological environment. YC@dye@SiO_2_ will be applied as a carrier in drug delivery and imaging for theranostic anticancer therapies. Furthermore, functional vinyl groups on the YC@dye@SiO_2_ surface are available for immobilizing various biological compounds for targeted therapies, such as antibodies and biotinylated reagents.

## Figures and Tables

**Table 1:** Quantum yields for the generation of singlet oxygen.

Compound	Solvent	Φ_Δ_	10^6^ Φ_P_
PSF	DMF	0.23	91.32
	DMSO	0.63	70.17
YC@PSF	DMF	0.0031	-
	DMSO	0.0023	-
YC@PSF@SiO_2_	DMF	0.0023	-
	DMSO	0.0238	-
ZnPc	DMF	0.56	10.2
	DMSO	0.67	3.5

## Data Availability

Data are contained within the article.
